# Racial Differences in the Biochemical Effects of Stress in Pregnancy

**DOI:** 10.3390/ijerph17196941

**Published:** 2020-09-23

**Authors:** Paris Ekeke, Dara D. Mendez, Toby D. Yanowitz, Janet M. Catov

**Affiliations:** 1 Department of Epidemiology, University of Pittsburgh Graduate School of Public Health and Neonatal-Perinatal Medicine, UPMC Magee Women’s Hospital, Pittsburgh, PA 15213, USA; 2 Department of Epidemiology, University of Pittsburgh Graduate School of Public Health, Pittsburgh, PA 15261, USA; ddm11@pitt.edu; 3 Division of Neonatal-Perinatal Medicine, UPMC Magee Women’s Hospital, Pittsburgh, PA 15213, USA; yanotd@upmc.edu; 4 Department of Obstetrics, Gynecology, & Reproductive Services, UPMC Magee Women’s Hospital and Department of Epidemiology, University of Pittsburgh Graduate School of Public Health, Pittsburgh, PA 15261, USA; catovjm@upmc.edu

**Keywords:** disparity, toxic stress, inflammation, cytokines, race

## Abstract

Prenatal stress has been linked to preterm birth via inflammatory dysregulation. We conducted a cross-sectional study on female participants who delivered live, singleton infants at University of Pittsburgh Medical Center Magee Women’s Hospital. Participants (*n* = 200) were stratified by cumulative risk scores using a combination of individual factors (maternal education, diabetes, hypertension, smoking, relationship status, obesity, depression) and neighborhood deprivation scores. We hypothesized that inflammatory cytokines levels differ by risk group and race. Multiplex analyses of IL-6, IL-8, IL-10, IL-13 and TNF-alpha were run. We found that Black birthing people had more risk factors for chronic stress and had lower levels of IL-6 compared to White birthing people. When stratified by risk group and race, low-risk Black birthing people had lower levels of IL-6 compared to low-risk White birthing people, and high-risk Black birthing people had lower levels of IL-8 compared to high-risk White birthing people. Higher area deprivation scores were associated with lower IL-6 levels. Our results suggest that the relationship between chronic stress and inflammatory cytokines is modified by race. We theorize that Black birthing people encounter repetitive stress due to racism and social disadvantage which may result in stress pathway desensitization and a blunted cytokine response to future stressors.

## 1. Introduction

Toxic stress refers to chronic exposure to physical, emotional, and/or environmental stressors that results in biochemical changes in the absence protective factors [1]. Exposure to repetitive negative exposures can be toxic to the body’s stress response and may lead to overstimulation of the hypothalamic-pituitary (HPA) axis and subsequent alterations along the stress pathway. Toxic stress can also have long term negative effects on the body, including decreased hippocampal volume, immune cell dysregulation, and altered DNA methylation patterns [2]. In regards to pregnancy, toxic prenatal stress has been postulated to be a factor contributing to preterm birth and low birth weight via an inflammatory pathway [3]. There is a progressive cascade involving maternal and fetal HPA activation that leads to a common pathway of parturition characterized by uterine contractility, cervical ripening and fetal membrane activation [4]. Although stress has been implicated in promoting the pathway to preterm birth, the immunomodulation involved in chronic exposure to stress does not appear to affect each individual in the same manner. It has been theorized that the interaction between medical risk factors and environmental exposures may explain the differential effects of chronic stress between races and their birth outcomes [5]. There is a persistent racial disparity in birth outcomes in the United States that disproportionately affects Black parents. The etiology of this disparity remains poorly understood, but many contributing factors have been identified, and repetitive exposure to negative stressors may play a role.

Stress may take on many different forms in the daily lives of individuals. A substantial body of literature has examined the effects of physical stressors, in the form of medical risk factors, on the racial disparity in preterm births. While Black people have been found to have a higher prevalence of hypertension and diabetes, when controlling for body mass index (BMI), diabetes mellitus, pregnancy-induced hypertension and eclampsia, the over-representation of Black birthing people in preterm birth rates remains [6,7]. Additionally, access to medical care has been historically considered a major risk factor for preterm birth. Some studies have reported lower prenatal utilization rates in Black people compared to other races, particularly early in pregnancy. This intentional underutilization of prenatal care has been suggested through patient interviews to be due in part to exposure to institutionalized racism and perceived bias when presenting to medical attention [8,9,10]. While later presentation to care may negatively impact maternal and infant outcomes, recent trends suggest the gap in prenatal care utilization is rapidly closing between races so access to care does not fully explain the disparities in birth outcomes [9].

In addition to an individual’s medical risk factors, social factors must also be considered as a source of stress and significant influence on one’s personal health. Socioeconomic status has often been considered a major contributor to the racial disparity in preterm birth. Education attainment is one method to improve one’s social position in society and access to resources. For Black parents, increases in maternal education attainment and income levels appear to not be as protective compared to other races. Racial disparities in preterm birth rates widen as education and income levels increase [11,12,13]. In low income groups, there is a similarly high rate of preterm births between races, but within the most socioeconomically advantaged group, there is a significant Black–White disparity in preterm birth rates. Well-educated Black people have higher preterm birth rates compared to White people of the same or lower educational status [13]. This alarming trend implores us to closely examine the unique experience of Black birthing people in the U.S. that may have a negative impact on their health outcomes. Review of the literature has identified several contributing factors, but controlling for individual-level maternal risk factors, such as chronic medical conditions, access to prenatal care, and socioeconomic status, does not fully explain the racial disparity seen in preterm births [5,14,15].

### Environmental Stress Also Contributes to the Disparity

It is hypothesized that the widening racial disparity can be attributed to a combination of individual and ecologic risk factors. Residents of deprived neighborhoods, characterized by violent crime, poverty, high vacancy rates and more housing damage have been associated with increased risk of preterm birth likely via a stress pathway [16,17,18]. In addition to the stress of being a resident of a deprived neighborhood, through isolation, the environment also can contribute to psychological stress. Lack of social support and use of avoidance coping mechanisms have been related to higher levels of depressive symptoms in pregnant women which is associated with increased risk for preterm birth, even after controlling for other sociodemographic and obstetric factors [19,20,21,22]. It has been suggested in previous studies that major depression in a susceptible host may lead to alterations in corticotropin-releasing hormone (CRH) levels which act at the placental interface propagating a cascade to preterm birth [23]. There is growing evidence that physical stress, environmental stress, and psychological stress alone explain portions of the racial disparity seen in preterm births, but to date, the cumulative effect remains unknown. Instead of focusing on individual stressors, there has been a shift toward examining how stressors accumulate and interact with each other. Currently, we are unable to identify in which individuals known stressors ultimately become toxic to one’s health and which stressors are most important. Because of this ambiguity, cumulative risk scores have been used in previous studies to attempt to capture aggregate effects of chronic stressors, but cumulative risk scores are limited by the quality of variables included in any given index.

Previous studies have identified a variety of other biomarkers for chronic stress with a focus on the inflammatory pathway. Dehydroepiandrosterone (DHEA), pro-inflammatory cytokines, corticotropin-releasing hormone (CRH), and tumor necrosis factor-alpha (TNF-alpha) have all been implicated to be part of the biophysical dysregulation in response to stress [22,24,25]. While patient reported stress scales provide subjective assessments of the severity, biochemical markers, such as cytokine levels, can be used to represent the downstream effects of chronic exposure to stress [24,25]. Wright et al. demonstrated increased levels of IL-8, IL-13, and TNF-alpha in birthing people with high prenatal stress scores resulting in alterations in innate and adaptive immune response in the cord blood of infants. This suggests that stress-induced immunomodulation in the in-utero environment leads to measurable biochemical changes in infants [25,26,27]. Previous studies have also noted that even in otherwise normal pregnancies, high levels of stress with low social support were associated with low levels of the anti-inflammatory cytokine, IL-10, early in pregnancy and higher levels of the pro-inflammatory cytokine, IL-6, later in pregnancy, highlighting the dynamic relationship between inflammation and pregnancy [28]. It is hypothesized that chronic exposure to stress disrupts the physiologic homeostasis between pro-inflammatory and anti-inflammatory cytokines and leads to inflammatory changes.

There is also literature to suggest that some individual risk factors for stress also are related to inflammation. Studies on pregnant women with hypertensive disorders, including pre-eclampsia have found associations with a pro-inflammatory state, characterized by elevations in IL-6 and TNF-alpha levels [29,30]. Elevated IL-6 and TNF-alpha, in addition to inflammatory markers, such as CRPs were also implicated in the pathogenesis of type 2 diabetes and gestational diabetes [31]. Although depression has not be traditionally thought to be associated with inflammation, a recent study by Achytes et al. identified several inflammatory changes that appeared to contribute to postpartum depression, including elevated IL-6 and IL-8 levels [32,33]. It also well known that obesity is associated with upregulation of certain pro-inflammatory markers. There are conflicting data on the relationship between smoking and inflammation, with some evidence for elevations in pro-inflammatory cytokines, but smoking may also induce suppression of the innate immune response [34]. Although previous literature has suggested that these individual stressors may lead to elevations in some pro-inflammatory cytokines, there is a paucity of data on the cumulative effect these factors have on inflammation during pregnancy.

To date, it has been established that cumulative prenatal stress, in the form of physical, environmental, and psychosocial stress, has a negative impact on preterm birth rates. The literature also supports that inflammation has a role in preterm birth [35,36]. Our study sets out to more closely examine the relationship between toxic stress and inflammation. While previous studies have primarily focused on the effects of singular risk factors, there are currently sparse data on the cumulative effect of personal and environmental factors on the stress pathway via inflammatory cytokines. We sought to close this gap and characterize multiple negative exposures via a cumulative risk stratification process across multiple domains. The main purpose of our study was to examine if those with high cumulative risk scores, as characterized by individual and environmental stressors, also have higher median levels of pro-inflammatory cytokines compared to low-risk individuals in the first trimester of pregnancy, which more closely represents pre-pregnancy stress levels.

## 2. Materials and Methods

**Figure 1:**
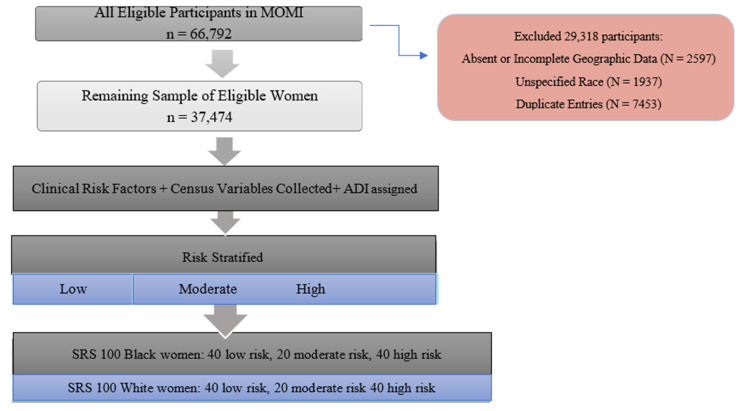
Sample Selection Process for Enrollment.

We conducted a cross-sectional study which was approved by the Institutional Review Board of University of Pittsburgh (IRB#PRO18080397). We enrolled 37,474 participants from the Magee Obstetric Maternal and Infant (MOMI) database aged 15–45 years old who delivered live, singleton infants during 2007–2013. MOMI is an electronic database of maternal and infant variables from all deliveries at Magee Women’s Hospital in Pittsburgh, PA. For the purposes of our study, participants were excluded if they did not have complete data for any of the pertinent risk factors, infant gestational age, or residential address. If a participant had multiple deliveries in the time period of interest, the first delivery was used for analysis and the remaining were excluded (Figure 1).

### 2.1. Individual-Level Variables

For each participant, the following variables were collected from the MOMI database: maternal education, race, BMI, history of chronic illness (defined as hypertension or diabetes), smoking status, marital status, history of depression, infant birth weight and gestational age. The residential address at time of delivery was extracted and geocoded into their corresponding census tracts.

### 2.2. Census Data

Individual income data were not available in the database, thus, census data at the tract level were used as a surrogate to capture financial status of each participant’s neighborhood. Socio-economic information from the United States Census Bureau was collected by census tract using 2009–2013 American Community Survey (ACS) 5-Year Estimates. Census tracts were used as a proxy for neighborhood. Estimates for the 8 census level variables that comprise the Messer Index were obtained: percent of males in management and professional occupations, percent of crowded housing, percent of households in poverty, percent of female headed households with dependents, percent of households on public assistance, and households earning < $30,000 USD per year estimating poverty, percent earning less than a high school education, and the percent unemployed.

### 2.3. Area Deprivation Index

**Figure 2:**
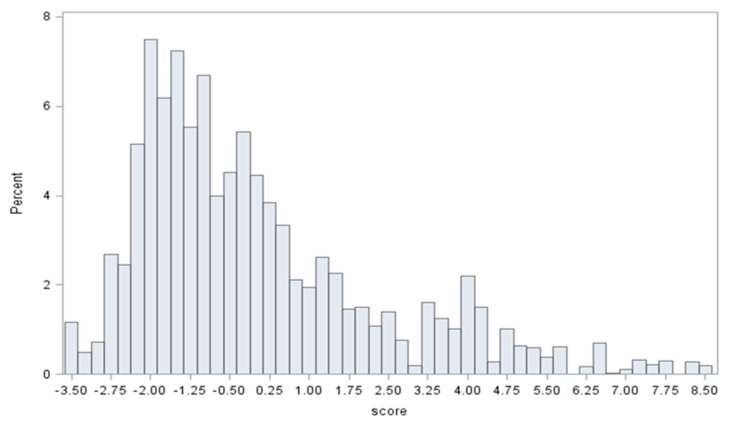
Distribution of area deprivation scores: Below is a histogram depicting the distribution Messer Area Deprivation Index (ADI) scores before tertiles were formed. Tertile 1(least deprived): ADI < −1.399. Tertile 2 (moderately deprived): −1.399 < ADI < 0.288. Tertile 3(most deprived): ADI > 0.288.

The Messer index was utilized because it has been used as a composite measure of area deprivation in previous studies on maternal and infant outcomes [37]. Using census tract data regarding their neighborhood, each woman was assigned a Messer Area Deprivation Index (ADI) score (Figure 2). Tertiles of the continuous neighborhood deprivation index score were created with higher percentiles indicating a higher degree of area deprivation. The tertiles were defined as low deprivation(<33%ile), moderate(33–66%ile), and high deprivation(>66%ile).

### 2.4. Risk Stratification of Cohort

Risk groups were assigned for the entire cohort as low, moderate, and high risk for toxic stress, which was assigned by the principal investigator based on the frequency of stressors/risk factors present. Risk factors for toxic stress included individual level characteristics defined as low maternal education (high school diploma or less), BMI > 25, smoking status of current smoker, relationship status of single, divorced, or widowed, history of chronic illness, and history of depression, in addition to living in a significantly deprived area, which was defined as having an area deprivation (ADI) score in the highest tertile (>66 percentile). Each risk factor was converted into a dichotomous variable and assigned zero if absent or one if present and a cumulative risk score was assigned to each participant.

Some of the inferences made about cumulative risk scores have been derived from studies on adverse childhood events(ACEs); cutoffs of at least 3 risk factors have been used, but there is no consensus [38]. Based on the distribution of the total number of risk factors per subject in our data, risk groups for toxic stress were formed using natural cut points. There were 39.5% (*n* = 14,818) in the low-risk group with 0–1 risk factors, 42.3% (*n* = 15,849) with 2 risk factors, and 18.2% (*n* = 6807) in the high-risk group with 3 or more risk factors. When risk stratification did not include ADI, there were 34.8% (*n* = 13,041) in the low-risk group, 57.3% (*n* = 21,473) in the moderate-risk group and 7.9% (*n* = 2960). Once the entire cohort was risk stratified using the cumulative risk score with ADI, a random sample was taken of 100 Black birthing people (40 high risk, 20 moderate risk, 40 low risk) and 100 White birthing people (40 high risk, 20 moderate risk, 40 low risk) for further analysis. Random sample was designated using a random number generator until the 100 women of each race were selected.

### 2.5. Cytokine Analysis

Maternal blood specimens were collected in the first trimester of pregnancy (mean gestational age 12.5 weeks). On each specimen, a panel of inflammatory markers (IL-6, IL-8, IL-10, IL-13, TNF-alpha) was run using multiplex fluorescent bead-based assay (Human High Sensitivity T Cell Magnetic Bead Panel). The observed concentration (pg/mL) of each analyte for each sample was calculated.

The distribution of each cytokine was assessed for normality using the Kolmogorov–Smirnov tests for normality. None of the cytokines had a normal distribution (*p* < 0.0001), therefore, nonparametric tests were performed for all subsequent analysis. IL-6 had a median (Q1, Q3) level of 10.1 (4.0, 23.3). IL-8 had a median (Q1, Q3) level of 12.6 (8.0, 31.9). IL-10 had a median (Q1, Q3) level of 34.9 (24.8, 34.9). IL-13 had a median (Q1, Q3) level of 7.9 (5.4, 14.5). TNF-alpha had a median of 8.1 (6.3, 10.6). Spearman correlation coefficients were calculated to assess each cytokine’s relationship with each other and with ADI scores.

### 2.6. Statistical Analysis

The primary aim was to determine if the participants with high cumulative risk scores had higher median cytokine levels compared to moderate- and low-risk groups. Median cytokine levels were calculated for each risk group. Wilcoxon rank sum and Kruskal–Wallis tests were performed to assess for differences in median cytokine levels stratified by risk group alone and race alone. Stratified analyses were performed by risk group and race to assess for (A) differences in median cytokine levels by risk group in White participant, (B) Black participants, (C) difference by race within the high-risk group, (D) difference by race within the moderate-risk group, and (E) difference by race within the low-risk group. Bonferroni corrections were used to account for multiple comparisons. Multivariable regression models were conducted to calculate the adjusted median cytokine levels, controlling for risk group, race, maternal age, parity, and BMI. The statistical package for analysis was SAS software, version 9.4 (SAS Institute Inc., Cary, NC, USA).

## 3. Results

Within the entire cohort (*n* = 37,424), there were differences in the baseline characteristics by race (Table 1). Black participants were younger (24.8 vs. 29.5 years, *p* < 0.0001), had higher pre-pregnancy BMI (27.4 vs. 25.4 kg/m^2^, *p* < 0.0001), were more likely to be nulliparous (36% vs. 29.3%, *p* < 0.0001) and were more likely to have hypertension (9.2% vs. 6.4%, *p* < 0.0001) and depression (11.1% vs. 8.8%, *p* < 0.0001) compared to White participants. They were less likely to be married (11.9% vs. 49.5%, *p* < 0.001) and less likely to smoke (12.3% vs. 16.0%, *p* < 0.0001) compared to White participants. Regarding birth outcomes, Black participants had higher preterm birth rates (12.2% vs. 9.0%, *p* < 0.0001), higher rates of small for gestational age (<10%ile for gestation) (16.4% vs. 8.3%, *p* < 0.0001) and their infants had lower birth weights (3097g vs. 3349g, *p* < 0.0001). In the high-risk group, 53/77 (68.8%) live in the most deprived area (ADI > 66%ile) compared to 15/39 (38.4%) in the moderate-risk group and 4/85 (4.7%) in the low-risk group that lived in a deprived area with a *p*-value of <0.0001.

In our stratified sample (*n* = 200), there were three significant differences noted between races. Black participants had higher rates of SGA infants (15% vs. 6%, *p* = 0.01) and lower rates of being in a committed relationship (29% vs. 48%, *p* = 0.01) compared to White participants. Black women were also younger (mean age 26.8 vs. 32.4) compared to White participants. There were no other significant racial differences in baseline characteristics of our stratified random sample (Table 2). Baseline characteristics stratified by risk group are depicted in Table 3. High-risk participants had higher rates of being single, having hypertension, smoking, having a HS diploma or less, having depression, and preterm birth compared to low-risk participants.

The results of the Spearman correlation coefficient cytokine matrix are depicted in Table 4. IL-6 was strongly correlated with IL-13 (R = 0.828, *p*-value < 0.001) and weakly correlated with IL-8 (R = 0.381, *p*-value < 0.0001) and IL-10 (R = 0.578, *p*-value < 0.0001). The remaining cytokines (IL-8, IL-10, IL-13, TNF-alpha) did not show any statistically significant correlations with each other. The relationship between each cytokine and ADI was assessed, and IL-6 had a weak negative correlation with area deprivation (R = −0.155, *p*-value = 0.029).

When stratified by risk group, there were no statistically significant differences in median levels of IL-6, IL-8, IL-10, IL-13, or TNF-alpha (Table 5). When stratified by race, Black participants were found to have lower median IL-6 levels (8.80 vs. 10.56, *p* = 0.037) compared to White participants (Table 6). Median IL-8, IL-10, IL-13, TNF-alpha levels were not significantly different between Black and White participants. When stratified by race and risk group, there were differences between median cytokine levels observed (Table 7), but some of these differences disappear when adjusting for BMI, parity, and maternal age (Table 8). Low-risk Black participants had lower median IL-6 levels (9.6 vs. 13.9, *p* = 0.02) and compared to low-risk White participants after controlling for BMI, parity and maternal age. High-risk Black women also had lower adjusted median IL-8 levels (12.1 vs. 18.9, *p* = 0.02). There were no statistically significant differences between moderate-risk Black and White participants. The participants in the most deprived area (ADI > 66%ile) had a lower median (Q1, Q3) IL-6 level of 8.53 (5.04, 18.64) compared to those in the moderately deprived with median of 11.59 (7.72, 30.75) and least deprived groups with a median of 9.52 (7.18, 23.20), *p*-value = 0.008.

Multivariable regression models showed that after controlling for maternal age, parity, and BMI, low-risk Black participants had lower median IL-6 levels compared to low-risk White participants (9.6 vs. 13.9, *p* = 0.02). High-risk Black participants had lower median IL-8 levels compared to high-risk White participants (12.1 vs. 18.9, *p* = 0.02). There were no other significant differences between race or risk groups when controlling for maternal age, parity, and BMI.

## 4. Discussion

Our study emphasizes the importance of race in the discussion of prenatal stress. Based on our results, there were no appreciable differences in median cytokine levels between participants with high cumulative risk scores compared to moderate and low risk. When considering race, we found that Black participants had lower IL-6 levels compared to White participants. Additionally, the relationship between cytokines and risk group appears to be race dependent. Even for low-risk pregnant people in the first trimester with lower cumulative stress scores, there were lower levels of IL-6 in Black participants compared to White participants. This suggests that even for participants with low levels of pre-pregnancy stress, there is a White–Black difference that is left unexplained. For high-risk pregnant participants in the first trimester, Black participants similarly had lower IL-8 levels compared to high-risk White participants. Although Black participants were younger with higher BMIs compared to White participants in the original cohort, which may affect levels of pro-inflammatory cytokines, there were no significant differences in these baseline variables in the analytic sample (*n* = 200). We suspect there are negative exposures not captured in our cumulative risk assessment that may impact inflammatory cytokine levels.

In addition to being influential in risk group delineation, ADI was also associated with biochemical changes. ADI was correlated with IL-6 in a slightly negative direction. As area deprivation increased, IL-6 levels decreased by 0.155. Although a modest effect, the negative correlation further supports our findings of a possible blunted inflammatory response in the setting of multiple stressors. Without the addition of ADI in the cumulative risk assessment, risk group designation would have been much more difficult. Initially, we observed a predominance of moderate-risk participants, but with addition of ADI in the cumulative risk assessment, many of the moderate-risk participants shifted to the high-risk group. We believe this provides further support that inclusion of area-level indices is influential in providing a more complete risk assessment. We theorize that chronic exposure to significant stressors may alter the baseline inflammatory milieu for Black birthing people, thus putting them at risk for inflammatory changes in the setting of a new stressor such as pregnancy.

Our study extends the current knowledge of inflammatory changes that may be associated with multiple stressors. While previous studies have found exposure to chronic stress could lead to elevation of pro-inflammatory cytokines, our results suggest there also can be a blunted stress response. Although not directly assessed in our study, we hypothesize that the unique experience of being a Black person in America with repeated negative exposures such as discrimination and microaggressions may be a source of chronic stress that could play a role in aberrations in cytokine levels resulting in lower inflammatory levels. Our current belief is that repetitive stress may play a role in desensitization of the stress pathway and the receptors involved, so in the face a new stressor, one is unable to mount a full, robust stress response. This phenomenon has been noted in previous studies. Ouellet-Morin examined the stress response of adolescents who were being chronically maltreated and exposed to chronic stress through bullying and found a similar effect. When compared to the control group, the chronically maltreated/bullied child had lower cortisol levels in response to additional psychosocial stress [39]. Additional studies have reported similar effects of decreased HPA axis reactivity in response to chronic stress among widows, teachers experiencing burnout, and healthy adults with history of moderate to severe childhood maltreatment [2,40,41,42,43]. While previous studies have documented this phenomenon in cortisol, our study extends current knowledge to include cytokines such as IL-6.

Even in the absence of many risk factors for stress, Black participants in our low-risk group had a differing IL-6 response compared to White women in a similar strata. Outside of risk groups formed by medical, sociodemographic, and environmental risk factors that are involved in repetitive activation of the stress response, race seemed to be influential in determining how those risk factors may alter inflammatory cytokines. It has been well documented that even in the most socioeconomically advantaged strata of Black people, workplace discrimination in the form of microaggressions, overt racism and sexism are chronic sources of stress, so it is possible that low-level chronic stressors not captured in our study may explain some of the racial difference we observed between low-risk groups [44,45]. Despite adequate access to medical care, there is a growing body of evidence that the quality of medical care Black people receive is often different than other races [46,47]. Research has pinpointed the combination of the structural racism of the health system as well as the unconscious bias of health professionals to be contributing to the disparity in adverse birth outcomes [48,49,50]. Black inferiority attitudes, lack of empathy, and discriminatory hospital practices also play a role in the inequity of care received by Black people [46,51]. As a result, stress has been posited as an intervening pathway between racism and preterm birth [52]. As individual risk factors accumulate in the high-risk group, Black birthing people are still being exposed to race-based discrimination and may experience burnout of their pro-inflammatory pathway, thus leading to lower IL-6 levels compared to White birthing people with similar individual risk factors. Disparities in preterm birth are likely not the result of the singular effect of any risk factor but are evidence of how medical and environmental stressors in combination with lifelong exposure to racism, sexism, and disadvantaged social status lay the foundation for HPA axis dysregulation.

Although our study adds additional context to the discussion of toxic stress, there were limitations. Assessment of cytokine levels and clinically significant differences was limited due to the retrospective design of the study. We were limited by the quality of the variables collected, so we are unable to comment on how the severity of risk factors, such as diabetes, hypertension or depression, contributes to the overall picture. Our sample only included participants with first trimester samples available and recorded a delivery of a live infant, so we cannot comment on the effect of toxic stress on miscarriages and on other factors that may impact outcomes, including late presentation to care. Since our sample represented only a specific subset of the entire cohort, we were able to focus on comparing cytokine levels at similar time points in a population with similar prenatal care utilization trends to minimize confounding. The cytokine levels assessed in this study merely provide a snapshot of the dynamic complexity of biochemical changes associated with stress. It is possible pro-inflammatory and anti-inflammatory changes are different at different time points prior to pregnancy and through each trimester of pregnancy. For this reason, we are not able to draw any definite conclusions about what effect the observed biochemical effects have on birth outcomes, particularly preterm birth, since first trimester samples were used and the primary goal of the study was to examine the relationship between stress and inflammation, not inflammation and preterm birth. We identified two additional limitations of our study which may explain why we were unable to detect a difference in median cytokine levels in participants with high cumulative stress scores compared to moderate and low-risk participants. First, the small sample size significantly limited the power of our study, and with larger numbers we may be able to more accurately assess the more subtle differences between risk groups and inflammation. Multiple comparison tests were performed, and although we attempted to correct for this in the statistical analysis, there is always a chance that the small changes detected are not of clinical significance. Secondly, it is possible that due to the complexity of the relationship of risk factors, the use of a cumulative risk score is insufficient. The relationship between race, inflammation and stress is dynamic, and individual risk factors may affect inflammatory cytokines differently, thus, by aggregating multiple risk factors, there is a chance that we washed out differences between groups. With a larger sample size, we would be able to look at more subtle differences between individual characteristics to make inferences about specific negative exposures and cytokine levels. Our study was exploratory and merely serves as an introduction into the ways race plays a role in inflammation in pregnancy, but more research is needed to confirm and expand upon our findings. Future studies are needed with sufficient power for multilevel modelling to further delineate which environmental and individual factors are the most significant contributors and how cytokine levels change closer to the time of delivery.

## 5. Conclusions

In summary, we know that chronic exposure to multiple stressors has short-term and long-term implications on maternal and infant health. While previous literature has shown individual stressors may lead to prolonged activation of the inflammatory pathway, our study suggests an interaction between race and risk factors for stress that could be implicated in stress propagation on the pathway to toxic stress. Our study extends the knowledge about the relationship between inflammation and stress to not only include elevations in pro-inflammatory cytokines, but also in a susceptible host, a blunted response in the pro-inflammatory cytokines that may affect the adaptive response of individuals to future stressors. Future prospective studies may be able to associate inflammatory changes seen by the cumulative effect of multiple stressors to preterm birth disparities. Next steps include studies to better understand the race-dependent biochemical differences associated with chronic stress and how the physiologic changes throughout pregnancy directly contribute to adverse birth outcomes.

## Figures and Tables

**Table 1:** Racial differences in baseline characteristics of the entire eligible cohort (*n* = 37,474).

	White *n* = 26,232	Black *n* = 8994	*p*-Value
Preterm	2360 (9.0%)	1097 (12.2%)	<0.0001
Small for Gestational Age(SGA)	2177 (8.3%)	1475 (16.4%)	<0.0001
Age	29.6 (SD 5.4)	24.8 (SD 5.7)	<0.0001
Nulliparity	7686 (29.3%)	3238 (36.0%)	<0.0001
Hypertension	1679 (6.4%)	827 (9.2%)	<0.0001
Diabetes	1653 (6.3%)	441 (4.9%)	<0.0001
Smoking	4194 (16.0%)	1070 (12.3%)	<0.0001
History of Depression	2308 (8.8%)	998 (11.1%)	<0.0001
Relationship Status (Committed, Married or Life Partner)	12,977 (49.5%)	875 (11.9%)	<0.0001
High School Diploma or less	9050 (34.5%)	5726 (63.7%)	<0.0001
Pre-pregnancy weight(kg)	68.6 (SD 16.8)	73.7 (20.2)	<0.0001
BMI	25.4 (SD 5.90)	27.4 (SD 7.1)	<0.0001
Birth weight(g)	3349 (SD 580)	3097 (SD 636)	<0.0001

**Table 2:** Racial differences in baseline characteristics of stratified sample (*n* = 200).

	White (%)	Black (%)	*p*-Value
Preterm	8	11	0.54
SGA	6	15	0.01 *
History of Depression	8	12	0.39
Hypertension	11	9	0.57
Diabetes	5	6	0.81
Smoking	13	13	0.99
Committed Relationship	48	29	0.01 *
High School Diploma or less	42	31	0.16
BMI > 25	6	3	0.28
Nulliparous	34	29	0.43
Age(Years)	32.4	26.8	0.03 *

* statistically significant with *p*-value < 0.005.

**Table 3:** Baseline characteristics of stratified sample (*n* = 200).

	Low Risk *n* = 85	Moderate Risk *n* = 39	High Risk *n* = 77	*p*-Value
BMI > 25	3 (3.5%)	2 (5.1%)	4 (5.2%)	0.88
Hx of Diabetes	2 (2.4%)	5 (12.8%)	4 (5.2%)	0.086
Single	9 (10.6%)	25 (64.1%)	44 (57.1%)	<0.0001 *
Hx of Hypertension	5 (5.9%)	3 (7.7%)	12 (15.6%)	<0.01 *
≤HS diploma	8 (9.4%)	24 (61.5%)	42 (54.6%)	<0.0001 *
Smoker	5 (5.9%)	3 (7.7%)	23 (29.9%)	<0.0001 *
Hx of Depression	1 (1.2%)	2(5.1%)	17 (22.1%)	<0.0001 *
Preterm Birth	6 (7.1%)	1 (2.6%)	12 (15.6%)	0.05 *
Nulliparity	12 (16.5%)	3 (7.7%)	17 (22.1%)	0.07
Mean Age (Years)	25.3	28.8	26.6	0.79

* statistically significant with *p*-value < 0.005.

**Table 4:** Cytokine correlation matrix.

	IL-6	IL-8	IL-10	IL-13	TNFA	ADI
IL-6	1.00	0.38	0.58	0.83	0.12	−0.16
*p*-value	--	<0.001 *	<0.001 *	<0.001 *	0.10	0.03 *
IL-8	0.38	1.00	0.13	0.30	0.18	0.01
*p*-value	<0.001 *	--	0.07	<0.001 *	0.01	0.85
IL-10	0.58	0.13	1.00	0.60	0.08	−0.10
*p*-value	<0.001 *	0.07	--	<0.001 *	0.28	0.16
IL-13	0.83	0.30	0.60	1.00	0.09	−0.10
*p*-value	<0.001 *	<0.001 *	<0.001 *	--	0.20	0.17
TNFa	0.12	0.18	0.08	0.09	1.00	−0.003
*p*-value	0.10	0.01	0.28	0.20	--	0.97

* statistically significant with *p*-value < 0.005.

**Table 5:** Median cytokine levels by risk group.

	Low Risk (0–1 Risk Factors) *n* = 85	Medium Risk (2 Risk Factors) *n* = 39	High Risk (3+ Risk Factors) *n* = 77	*p*-Value
IL-6	10.0 (6.6, 23.3)	8.9 (6.2, 21.2)	10.6 (6.5, 23.3)	0.91
IL-8	12.9 (8.7, 30.5)	13.4 (9.0, 36.2)	12.2 (7.9, 32.3)	0.75
IL-10	35.9 (25.4, 59.1)	34.9 (19.3, 51.1)	34.6 (26.1, 48.6)	0.63
IL-13	7.8 (5.6, 14.1)	7.6 (5.3, 16.1)	8.0 (5.3, 14.5)	0.87
TNF-a	8.1 (6.1, 10.4)	7.7 (6.0, 10.8)	8.1 (6.7, 10.8)	0.63

**Table 6:** Median cytokine levels stratified by race.

	Black Women Median (p25, p75)	White Women Median (p25, p75)	*p*-Value
IL-6	8.8 (5.3, 25.5)	10.6 (7.5,23.3)	0.04 *
IL-8	13.6 (8.0, 40.2)	12.2 (8.0, 24.6)	0.43
IL-10	36.0 (25.2, 47.7)	34.9 (23.8, 58.0)	0.39
IL-13	7.7 (5.2, 15.5)	8.3 (5.6, 14.5)	0.35
TNF-a	7.7 (6.1, 10.4)	8.5 (6.4, 10.8)	0.39

* statistically significant with *p*-value < 0.005.

**Table 7:** Median cytokine levels stratified by risk and race.

	Low Risk (0–1 Risk Factors) *n* = 85	Medium Risk (2 Risk Factors) *n* = 39	High Risk (3+ Risk Factors) *n* = 77	*p*-Value ^1^
IL-6				
White	10.3 (7.6, 20.5)	8.2 (6.4, 17.7)	13.9 (8.6, 26.4)	0.40
Black	8.9 (5.7, 25.4)	8.9 (5.8, 26.9)	7.9 (4.2, 19.8)	0.90
*p*-value ^2^	*p* = 0.24	*p* = 0.79	*p* = 0.05 *	
IL-8				
White	10.2 (7.5, 30.0)	12.3 (9.0, 46.4)	12.7 (8.6, 20.5)	0.45
Black	15.2 (10.8, 30.5)	13.6 (10.5, 28.4)	9.7 (7.0, 59.4)	0.43
*p*-value ^2^	*p* = 0.06	*p* = 0.57	*p* = 0.33	
IL-10				
White	42.3 (28.9, 67.6)	28.4 (16.3, 58.0)	32.1 (22.1, 49.4)	0.11
Black	31.5 (22.8, 47.0)	37.7 (20.8, 45.2)	38.4 (26.9, 48.6)	0.29
*p*-value ^2^	*p* = 0.02 *	*p* = 0.57	*p* = 0.33	
IL-13				
White	8.5 (5.8, 14.0)	7.4 (5.2, 13.5)	8.3 (6.6, 14.5)	0.68
Black	7.40 (5.0, 14.1)	7.8 (5.7, 16.1)	7.9 (4.8, 15.8)	0.98
*p*-value ^2^	*p* = 0.35	*p* = 0.83	*p* = 0.51	
TNFa				
White	8.5 (6.5, 11.1)	8.5 (6.0, 10.3)	8.4 (6.7, 10.4)	0.96
Black	7.7 (5.9, 9.0)	7.4 (6.3, 10.8)	7.8 (6.7, 11.3)	0.34
*p*-value ^2^	*p* = 0.19	*p* = 0.78	*p* = 0.98	

^1^*p*-value associated with testing for differences in median cytokine levels between risk groups within one race (Black or White) with Bonferroni correction. ^2^*p*-value associated with testing for differences between races within one risk group with Bonferroni correction. * statistically significant with *p*-value < 0.005.

**Table 8:** Multivariable regression model with adjusted cytokine medians ^‡^.

	Low Risk (0–1 Risk Factors) *n* = 85	Medium Risk (2 Risk Factors) *n* = 39	High Risk (3+ Risk Factors) *n* = 77	*p*-Value ^1^
IL-6				
White	13.9 (SE 2.2)	17.2 (SE 7.2)	16.3 (SE 6.9)	0.52
Black	9.6 (SE 1.5)	16.6 (SE 8.9)	16.2 (SE 8.1)	0.35
*p*-value ^2^	*p* = 0.02 *	*p* = 0.79	*p* = 0.99	
IL-8				
White	11.4 (SE 3.4)	18.6 (SE 5.8)	18.9 (SE 2.2)	0.45
Black	11.6 (SE 2.7)	18.0 (SE 5.2)	12.1 (SE 3.2)	0.78
*p*-value ^2^	*p* = 0.75	*p* = 0.98	*p* = 0.02 *	
IL-10				
White	43.0 (SE 16.2)	44.1 (SE 8.8)	56.4 (SE 16.7)	0.82
Black	62.8 (SE 19.5 )	63.8 (SE 8.6)	74.8 (SE 6.6)	0.91
*p*-value ^2^	*p* = 0.20	*p* = 0.09	*p* = 0.40	
IL-13				
White	6.6 (SE 8.8)	8.1 (SE 4.7)	7.3 (SE 3.6)	0.79
Black	4.2 (SE 10.7)	7.8 (SE 4.7)	7.2 (SE 3.8)	0.34
*p*-value ^2^	*p* = 0.26	*p* = 0.93	*p* = 0.81	
TNFa				
White	10.9 (SE 2.2)	13.2 (SE 1.2)	10.1 (SE 0.9)	0.12
Black	14.3 (SE 2.6)	15.8 (SE 1.4)	13.7 (SE 0.8)	0.84
*p*-value ^2^	*p* = 0.36	*p* = 0.09	*p* = 0.06	

^‡^ Median cytokine levels adjusted for BMI, parity, and maternal age. ^1^ *p*-value associated with testing for differences in median cytokine levels between risk groups within one race (Black or White) with Bonferroni correction. ^2^ *p*-value associated with testing for differences between races within one risk group with Bonferroni correction. * statistically significant with *p*-value < 0.005.
